# AKI in Hospitalized Children: Poorly Documented (and Underrecognized)

**DOI:** 10.3389/fped.2021.790509

**Published:** 2022-01-10

**Authors:** Katherine Jones, Alicia Neu, Jeffrey Fadrowski

**Affiliations:** Division of Pediatric Nephrology, Johns Hopkins School of Medicine, Baltimore, MD, United States

**Keywords:** acute kidney injury, electronic health record, pediatrics, nephrology, creatinine

## Abstract

**Background:** Acute kidney injury (AKI) is common in hospitalized children. We hypothesized that hospital-acquired AKI would be underrecognized and under-reported, with potential implications for prevention of future AKI and CKD risk stratification.

**Methods:** Five hundred thirty-two cases of AKI occurring over a 1 year period in a tertiary children's hospital in the United States were studied. AKI documentation was defined as any mention of AKI in the admission history and physical note, progress notes, or discharge summary. Nephrology follow-up was defined as a completed outpatient clinic visit within 1 year of discharge. Logistic regression was used to assess factors associated with documentation, consultation, and follow-up.

**Results:** AKI developed during 584/7,640 (7.6%) of hospitalizations: 532 cases met inclusion criteria. Documentation was present in 34% (185/532) of AKI cases and 90 (16.9%) had an inpatient nephrology consult. Among 501 survivors, 89 (17.8%) had AKI in their hospital discharge summary and 54 had outpatient nephrology follow up. Stage 3 AKI, peak creatinine >1 mg/dL and longer length of stay were associated with documentation. Stage 3 AKI and higher baseline creatinine were associated with inpatient nephrology consultation. Inpatient nephrology consultation was positively associated with outpatient nephrology follow up, but documentation in the discharge summary was not.

**Conclusion:** Most cases of AKI were not documented and the proportion of children seen by a nephrologist was low, even among those with more severe injury. Increased severity of AKI was associated with documentation and inpatient consultation. Poor rates of documentation has implications for AKI recognition and appropriate management and follow up.

## Introduction

Acute kidney injury (AKI) is common in hospitalized children (1) and the incidence is increasing (2). AKI is associated with increased length of stay and increased mortality among those who are critically ill (3). Children who experience AKI are at increased risk for hypertension, proteinuria, repeat AKI episodes, and chronic kidney disease (CKD) (4, 5).

Given its significant health impacts, the KDIGO (Kidney Disease: Improving Global Outcomes) guideline on AKI recommends evaluating patients for CKD 3 months after AKI (6). Nephrology follow-up has been associated with improved outcomes after severe kidney injury in adults (7), but outpatient nephrology follow-up after AKI in pediatric patients is uncommon (8, 9). This lack of appropriate follow-up is driven in part by a lack of AKI recognition (10). Understanding the scope of and clinical factors associated with AKI underrecognition in children is critical to developing strategies to improve detection and the management of those affected. Previous studies, including one of children in the intensive care unit, have shown that billing/administrative data has a low sensitivity in identifying AKI compared to KDIGO creatinine-based criteria, however, the extent of underrecognition of AKI by clinicians, was not described (11, 12). Documentation of AKI in the electronic health record (EHR) is likely a more sensitive surrogate for the clinical detection of AKI and therefore was the outcome we chose to study.

To further characterize recognition of AKI by clinicians, a detailed review of the EHR for documentation of AKI and nephrology consults was performed in hospitalized children with AKI based on KDIGO-creatinine criteria. We predicted that AKI would be poorly recognized and that the severity of AKI would be associated with an increased likelihood of documentation and nephrology consultation/follow-up.

## Methods

### Study Design and Data Sources

Among all hospitalized children admitted to a tertiary care pediatric hospital in the United States from July 1, 2016 through June 30, 2017, we conducted a retrospective chart review of the children with AKI. Patients >21 years of age, those admitted or discharged from the nephrology service, and patients with Stage 5 CKD treated with dialysis or transplant were excluded from analysis. AKI was identified in pediatric inpatients using a dashboard that interfaces with the EHR, developed to implement a patient safety program known as NINJA (Nephrotoxic Injury Negated by Just-In-Time Action). NINJA is a multi-center quality improvement collaborative with the goal of reduction in the incidence and intensity of AKI from nephrotoxic medications in hospitalized children (13, 14). Among identified cases of AKI, a manual review of the EHR was performed for demographic and clinical data, and assessment of documentation of AKI, nephrology consultation, and nephrology follow-up. Data were de-identified prior to data analysis. This study was approved by the institutional review board.

### Definitions

AKI was defined using KDIGO criteria for serum creatinine: an increase in serum creatinine from baseline by 0.3 mg/dL or >50% increase (6). AKI was staged based on peak serum creatinine using KDIGO criteria: Stage 1 (creatinine 1.5 to 1.9 times baseline or increase in serum creatinine ≥0.3), Stage 2 (creatinine 2 to 2.9 times baseline), and Stage 3 (creatinine ≥3 times baseline or dialysis). Urine output data was not collected for all patients and thus not used for AKI diagnosis or staging. Baseline creatinine was defined as the lowest creatinine measurement in the 6 months prior to the AKI episode. If no creatinine in the 6 months prior was available, the lowest creatinine during the hospitalization defined the baseline. AKI episodes were considered documented if there was any mention of AKI in the EHR. The following terms were used to search the EHR: AKI, kidney injury, kidney failure, acute renal failure, renal failure, renal injury, and renal insufficiency. AKI was counted in the patient's problem list if AKI or any of the other synonyms listed above were included in the patient's problem list during hospital stay or within 6 months after hospital discharge. Nephrology inpatient consultation was identified by presence of consult note documentation. Individuals were considered to have nephrology follow-up if they had a completed nephrology clinic visit within 12 months after hospital discharge.

### Analysis

All analyses were completed using Stata software version 16 (StataCorp, College Station, TX). Descriptive statistics were used to describe patient characteristics and prevalence of documentation, and nephrology consultation and follow-up. Multiple logistic regression was performed to determine associations between patient characteristics and the outcomes of interest (documentation, inpatient nephrology consultation, and outpatient nephrology follow-up).

## Results

Among 7,640 hospitalizations during the 1 year study period, 584 (7.6%) were complicated by AKI. Of the 614 patients identified as potential AKI cases by the EHR, 30 were either admitted for kidney transplant or receiving chronic dialysis and were thus excluded. An additional 52 patients were admitted to the nephrology service and also excluded. Of the 532 cases included, 148 (28%) were Black, 297 (56%) White, and 87 (16%) had race identified as other or not specified; 43 (8%) of patients were Hispanic. The median age was 11 years [interquartile range (IQR) 3–17 years]. The majority of the AKI events (51.5%) were KDIGO Stage 1 AKI.

Of hospitalizations complicated by AKI, 34.8% (185/532) had AKI documented in the EHR (Table 1). AKI was most often documented in the daily progress note. There were 31 deaths in the study period. Of those who died, 24/31 had Stage 3 AKI and 15/31 received dialysis while hospitalized. Of the deceased with Stage 3 AKI, 20/24 had a nephrology consult. Of the 501 surviving patients, 17.8% (89) had AKI documented in the hospital discharge summary. A minority of patients (16.9%) were seen by a nephrologist in the hospital, although this proportion increased to 52.5% among those with Stage 3 AKI. Similarly, outpatient nephrology follow-up was uncommon in the cohort (10.8% of all survivors) but more likely in those with more severe kidney injury (20.8% in those with Stage 3 AKI). Among the 29 (5.5%) patients who required acute dialysis, 14 survived to discharge; 21% (3/14) of these patients were seen in outpatient follow-up by nephrology.

**Table 1:** Summary of AKI documentation, nephrology consultation, and follow-up.

	**AKI (any stage)**	**Stage 1 AKI**	**Stage 2 AKI**	**Stage 3 AKI**
AKI events, % of total (*n*)	(532)	51.5% (274)	29.5% (157)	18.9% (101)
Documented in EHR, %	34.8%	22.6%	29.3%	77.2%
Discharge summary*	17.8%	10.4%	14.9%	49.4%
Progress note	32.1%	19.3%	25.5%	77.2%
History & physical	8.4%	5.5%	5.1%	21.8%
Documented in Problem List*, %	19.8%	14.1%	18.2%	43.4%
Nephrology inpatient consult, %	16.9%	8%	9.5%	52.5%
Nephrology outpatient follow-up*, %	10.8%	7.8%	11%	20.8%

*EHR, electronic health record*.

* *Among patients surviving to discharge (n = 501)*.

Duration of AKI was assessed for those patients with Stage 3 AKI. Median duration of injury was 7 days (IQR 3–16 days). After excluding patients with transient AKI (3 days or less), 65% had inpatient nephrology consultation. Among survivors of Stage 3 AKI lasting 4 days or more, 25% (11/44) had outpatient nephrology follow up.

Patient characteristics associated with AKI documentation in adjusted analyses included Stage 3 AKI, peak creatinine >1 mg/dL, and increased length of stay (Table 2). Table 3 shows the odds of inpatient nephrology consultation among those patients with AKI documented in the EHR. Patients with Stage 3 AKI and higher baseline creatinine were more likely to have nephrology consultation.

**Table 2:** Odds of AKI documentation by patient characteristic (*n* = 532).

	**OR (95% CI)**	***P***
Stage 1 AKI	1.00 (ref)	–
Stage 2 AKI	1.12 (0.59–2.13)	0.72
Stage 3 AKI	5.76 (2.58–12.88)	<0.001
Age (0–1)	1.00 (ref)	–
Age (2–13)	1.24 (0.62–2.49)	0.54
Age (14–21)	1.26 (0.62–2.54)	0.52
Baseline serum creatinine, by tertile*	0.87 (0.51–1.47)	0.59
Peak creatinine >1 mg/dL	8.00 (3.99–16.01)	<0.001
Length of stay, per day	1.007 (1.001–1.013)	0.03
ICU stay (Yes/No)	1.44 (0.86–2.43)	0.16

*Odds Ratio [OR] adjusted for all variables in table*.

* *Lowest serum creatinine in previous 6 months, baseline creatinine tertiles < 0.5, 0.5–0.6, and >0.6 mg/dL*.

**Table 3:** Odds of nephrology consultation by patient characteristic among those with documented AKI (*n* = 186).

	**OR (95% CI)**	***P***
Stage 1 AKI	1.00 (ref)	–
Stage 2 AKI	1.28 (0.45–3.63)	0.64
Stage 3 AKI	10.42 (3.46–31.35)	<0.001
Age (0–1)	1.00 (ref)	–
Age (2–13)	0.95 (0.34–2.65)	0.92
Age (14–21)	0.61 (0.23–1.59)	0.31
Baseline creatinine (by tertile)*	2.31 (1.19–4.46)	0.01
Peak creatinine > 1mg/dL	1.41 (0.58–3.45)	0.45
Length of Stay, per day	1.004 (0.99–1.01)	0.14
ICU Stay (Yes/No)	1.38 (0.60–3.19)	0.44

*Odds Ratio [OR] adjusted for all variables in table*.

* *Lowest serum creatinine in previous 6 months, baseline creatinine tertiles < 0.5, 0.5–0.6, and >0.6 mg/dL*.

There was a strong positive association between inpatient nephrology consultation and outpatient nephrology follow-up (OR 12.32, 95% CI 4.35–34.97) (Table 4). In contrast, there was no association between severity of AKI and nephrology follow-up. There was also no association between nephrology follow-up and documentation of AKI in the discharge summary.

**Table 4:** Odds of nephrology follow-up by patient characteristic among survivors with documented AKI (*n* = 163).

	**OR (95% CI)**	***P***
Stage 1 AKI	1.00 (ref)	–
Stage 2 AKI	1.40 (0.39–5.06)	0.61
Stage 3 AKI	0.99 (0.23–4.36)	0.99
Age (0–1)	1.00 (ref)	–
Age (2–13)	3.08 (0.72–13.09)	0.13
Age (14–21)	1.64 (0.38–7.01)	0.50
Baseline creatinine, by tertile*	1.22 (0.53–2.82)	0.64
Peak creatinine >1 mg/dL	1.67 (0.51–5.51)	0.40
Length of stay	0.99 (0.98–1.01)	0.21
ICU Stay (Yes/No)	1.09 (0.39–3.07)	0.87
Nephrology consulted	12.32 (4.35–34.97)	<0.001
Documentation in DC Summary	0.52 (0.19–1.40)	0.20

*Odds Ratio [OR] adjusted for all variables in table*.

* *Lowest serum creatinine in previous 6 months, baseline creatinine tertiles < 0.5, 0.5–0.6, and >0.6 mg/dL*.

There were 84 individuals who had >1 hospitalization complicated by AKI. This group accounted for 242/532 (45%) of all AKI episodes in the study. Those with multiple hospitalizations complicated by AKI were not more or less likely to have documentation of AKI, nephrology consultation or nephrology follow-up compared to those with a single hospitalization.

The discharge service with the majority of hospitalizations complicated by AKI in the study was oncology/bone marrow transplant (BMT) (41%), followed by general pediatrics (15%). The proportion of AKI documented and proportion of patients by discharge service is shown in Table 5. There was a decreased odds of AKI documentation in patients discharged by surgery services (OR 0.22, 95% CI 0.08–0.67) and by oncology/BMT services (OR 0.45, 95% CI 0.22–0.89). The proportion of those who had inpatient nephrology consultation is also described in Table 5. There was decreased odds of nephrology consultation in those discharged by cardiology (OR 0.24, 95% CI 0.06–0.91), NICU (OR 0.06, 95% CI 0.01–0.43), and oncology/BMT services (OR 0.15, 95% CI 0.04–0.51).

**Table 5:** Odds of AKI documentation and nephrology consultation by discharge service.

	**Proportion of AKI documented (%)**	AKI documentation adjusted OR* (95% CI)	**Proportion of patients with nephrology consult^¥^ (%)**	Nephrology consultation adjusted OR*^¥^ (95% CI)
General pediatrics	29/71 (41)	1.00 (ref)	20/30 (67)	1.00 (ref)
Cardiology	23/43 (53)	1.65 (0.67–4.02)	8/23 (35)	0.24 (0.06–0.91)
Surgery^‡^	8/48 (17)	0.22 (0.08–0.67)	4/8 (50)	0.27 (0.04–2.12)
PICU	25/42 (59)	0.57 (0.19–1.62)	20/25 (80)	0.65 (0.13–3.24)
NICU	15/41 (37)	0.49 (0.17–1.39)	5/15 (33)	0.06 (0.01–0.43)
Gastroenterology	14/36 (39)	0.50 (0.18–1.37)	8/14 (57)	0.62 (0.04–2.96)
Oncology/BMT	51/197 (26)	0.45 (0.22−0.89)	12/51 (24)	0.15 (0.04–0.51)

* *Multivariate model including discharge service, AKI stage, age, baseline creatinine, peak creatinine, and length of stay*.

¥ *Among those with documented AKI*.

‡ *Includes general pediatric surgery, neurosurgery, orthopedics, otolaryngology, and plastic surgery*.

Among patients who had AKI documented in the EHR and survived to hospital discharge, we found a lower odds of nephrology follow-up in those discharged by the oncology/BMT service (OR 0.24, 95% CI 0.07–0.85) in the multivariate model. Of note, 25 patients with documented AKI were discharged from the PICU (16 patients discharged as deceased and 9 survivors). Of the surviving patients discharged from the ICU, none completed outpatient nephrology follow-up.

## Discussion

In this study of children admitted to a tertiary care center over a 1 year period, AKI was common, with 7.6% of admissions complicated by AKI diagnosed by serum creatinine level, and almost half of the AKI events were Stage II or III severity. However, documentation of AKI in the EHR, including discharge summaries, progress notes and problem lists, was uncommon. Only a third of affected patients had AKI documented anywhere in the EHR; the proportion of documented cases increased as AKI severity increased. Approximately half of patients with Stage 3 AKI had an inpatient nephrology consult; only 20% of Stage 3 patients completed outpatient nephrology follow-up within 1 year. The factor most strongly associated with outpatient follow-up was inpatient consultation.

The incidence and the severity of AKI in this study is consistent with other studies of AKI among hospitalized pediatric patients (1, 2, 10). A few studies have demonstrated the poor sensitivity of billing/discharge codes in identifying AKI in pediatric patients, both in the US (15) and internationally (10, 16). Schaffzin et al. examined a cohort of non-ICU pediatric patients exposed to nephrotoxins who developed AKI and found that billing codes identified <50% of AKI episodes (15). Coding data has also been shown to have low sensitivity (17%) for AKI in a large adult cohort as well (11). This study is unique given the comprehensive review of the entire medical record for any mention of AKI and only 34% of all cases were documented. Our findings are consistent with what has been observed in other studies of pediatric AKI documentation. In the National Health Service in the United Kingdom, Bhojani et al. found that only 26% of AKI episodes were documented by the medical team based on a review of progress notes from a sample of 11% of patients with AKI (10). Menon et al. found that 47% of laboratory-identified kidney injuries were documented in a review of progress notes (17). We found that clinicians were more likely to document kidney for higher absolute values of peak creatinine (serum creatinine >1 mg/dL) as well as higher baseline creatinine suggesting that AKI is more readily recognized in pediatric patients who are closer to adult size. We also found that recurrent AKI was common in our cohort, but that individuals with recurrent AKI were no more likely to have their kidney injury documented or to receive nephrology consultation or follow-up compared to those with one AKI episode.

With regard to the long-term sequelae of AKI, a meta-analysis by Greenberg et al., found the incidence of CKD after AKI to be 3.7 per 100 person years (4). In a study of adult patients with severe AKI requiring temporary dialysis, early nephrology follow-up (within 90 days of hospital discharge) was associated with a decrease in mortality (7), although to date there is no study in a pediatric population correlating nephrology follow-up with outcomes. Nephrology follow-up after AKI is not routine in children (8, 9), perhaps due to a lack of recognition or the competing needs of other chronic medical conditions requiring intensive follow-up. A single center NICU cohort study found 40% of very low birth weight infants experienced AKI but none of them were referred for AKI follow-up and only 13.5% of survivors had their kidney injury documented in the discharge summary (9). In our study, even patients with severe kidney injury (Stage 3) had low rates of outpatient nephrology follow-up, even when their injury was documented in the EHR or discharge summary. Although AKI rates were among the highest for patients on the oncology/BMT service, these patients had decreased odds of outpatient nephrology follow-up compared to other services, even when accounting for severity of AKI.

Our findings add to the current body of literature describing underrecognition of AKI in hospitalized children. If AKI is not identified in the acute setting, important interventions such as medication dose adjustments and avoidance of nephrotoxins may not happen with implications for worsened severity of AKI and patient harm. Lack of recognition could result in missed opportunities for future AKI mitigation and missed screening for long-term sequelae such as hypertension, proteinuria, and CKD. Although nephrology follow-up may not be required for every episode of mild AKI, pediatric nephrologists are likely best suited to screen for and identify these post-AKI complications in children.

A proposed solution to the problem of underrecognition of kidney injury has been the use of provider alerts. In a single center study of AKI alerts in hospitalized children, Menon et al. implemented an alert and clinical decision support tool and showed improved documentation (<50–74% documented) and adjustment/discontinuation of medications. However, there was no statistical difference in documentation of AKI in the hospital discharge summary, nephrology consultation, or outpatient nephrology follow-up, perhaps due to small sample size (17). In a large, multicenter randomized controlled clinical trial of provider AKI alerts in adults, there was no difference in progression of AKI, dialysis or death between the intervention and control arms. Interestingly, there was an increased risk of death associated with AKI alerts in a sub-analysis of non-teaching hospitals (18). This finding deserves further study in the pediatric population, especially given the differences in AKI incidence, etiology, and comorbidities in children compared to adults. We propose the automated addition of AKI to the hospital discharge summary and/or problem list as a way to improve CKD screening, nephrology follow-up rates, and potentially mitigate severity and future risk of AKI.

Our study has several limitations. First, urine output was not strictly recorded for all patients and thus was not used to identify AKI, leading an underestimation of the true incidence. This study also described the consultation and referral practices at a single academic center and there may be variation in these practices depending on access to nephrology care, thus limiting generalizability. Also, patients receiving follow-up nephrology care at a different medical center with different EHR software were not captured. Finally, lack of documentation of AKI may not always indicate lack of recognition of AKI. The strengths of this study include the use of detailed chart and laboratory review rather than billing data leading to an increase in sensitivity and specificity of AKI documentation.

In conclusion, despite a significant incidence of AKI in hospitalized children, this study showed that most cases of AKI were not documented in the EHR, supporting limited existing literature that AKI is commonly underrecognized in hospitalized children. The proportion of children who received nephrology consultation and outpatient follow-up was also low, even among those with Stage 2 or 3 AKI. Additional work is needed to determine whether EHR alerts/automated problem list addition impacts the recognition and management of children with AKI and improves CKD screening and outcomes in AKI survivors. Future studies should assess whether EHR or clinical prompting of inpatient nephrology consultation and outpatient nephrology follow up among those with severe persistent AKI would improve screening for the sequela in affected children and decrease the risk of AKI recurrence.

## Data Availability Statement

The raw data supporting the conclusions of this article will be made available by the authors, without undue reservation.

## Ethics Statement

The studies involving human participants were reviewed and approved by Johns Hopkins Institutional Review Board. Written informed consent for participation was not provided by the participants' legal guardians/next of kin because: due to retrospective nature of the study and large sample size, consent was waived.

## Author Contributions

KJ, AN, and JF contributed to conception and design of the study. KJ compiled and organized the database and wrote the first draft of the manuscript. KJ and JF performed the statistical analysis. All authors wrote sections of the manuscript, contributed to manuscript revision, read, and approved the submitted version.

## Conflict of Interest

The authors declare that the research was conducted in the absence of any commercial or financial relationships that could be construed as a potential conflict of interest.

## Publisher's Note

All claims expressed in this article are solely those of the authors and do not necessarily represent those of their affiliated organizations, or those of the publisher, the editors and the reviewers. Any product that may be evaluated in this article, or claim that may be made by its manufacturer, is not guaranteed or endorsed by the publisher.

## References

[1] Sutherland SM Byrnes JJ Kothari M Longhurst CA Dutta S Garcia P. AKI in hospitalized children: Comparing the pRIFLE, AKIN, and KDIGO definitions. Clin J Am Sock Nephrol. (2015) 10:554–61. 10.2215/CJN.01900214 25649155 PMC4386245

[2] Parikh RV Tan TC Salyer AS Auron A Kim PS Ku E. Community-based epidemiology of hospitalized acute kidney injury. Pediatrics. (2020) 146:e20192821. 10.1542/peds.2019-2821 32784225 PMC7461200

[3] Kaddourah A Basu RK Bagshaw SM Goldstein SL. Epidemiology of acute kidney injury in critically ill children and young adults. N Engl J Med. (2017) 376:11–20. 10.1056/NEJMoa1611391 27959707 PMC5322803

[4] Greenberg JH Coca S Parikh CR. Long-term risk of chronic kidney disease and mortality in children after acute kidney injury: a systematic review. BMC Nephrol. (2014) 15:184. 10.1186/1471-2369-15-184 25416588 PMC4251927

[5] Mammen C Al Abbas A Skippen P Nadel H Levine D Collet JP. Long-term risk of CKD in children surviving episodes of acute kidney injury in the intensive care unit: a prospective cohort study. Am J Kidney Dis. (2012) 59:523–30. 10.1053/j.ajkd.2011.10.048 22206744

[6] Khwaja A. KDIGO clinical practice guideline for acute kidney injury. Kidney Int Supple. (2012) 2:19–36. 10.1038/kisup.2011.32 24571801

[7] Harel Z Wald R Bargman JM Mamdani M Etchells E Garg AX. Nephrologist follow-up improves all-cause mortality of severe acute kidney injury survivors. Kidney Int. (2013) 83:901–8. 10.1038/ki.2012.451 23325077

[8] Greenberg JH Zappitelli M Devarajan P Thiessen-Philbrook HR Krawczeski C Li S. Kidney outcomes 5 years after pediatric cardiac surgery: the TRIBE-AKI study. JAMA Pediatr. (2016) 170:1071–8. 10.1001/jamapediatrics.2016.1532 27618162 PMC5476457

[9] Carmody JB Swanson JR Rhone ET Charlton JR. Recognition and reporting of AKI in very low birth weight infants. Clin J Am Soc Nephrol. (2014) 9:2036–43. 10.2215/CJN.05190514 25280497 PMC4255405

[10] Bhojani S Stojanovic J Melhem N Maxwell H Houtman P Hall A. The incidence of paediatric acute kidney injury identified using an AKI E-alert algorithm in six english hospitals. Front Pediatr. (2020) 8:29. 10.3389/fped.2020.00029 32117834 PMC7026188

[11] Grams ME Waikar SS MacMahon B Whelton S Ballew SH Coresh J. Performance and limitations of administrative data in the identification of AKI. Clin J Am Soc Nephrol. (2014) 9:682–89. 10.2215/CJN.07650713 24458075 PMC3974361

[12] D'Arienzo D Hessey E Ali R Perreault S Samuel S Roy L. A validation study of administrative health care data to detect acute kidney injury in the pediatric intensive care unit. Can J Kidney Health Dis. (2019) 6:2054358119827525. 10.1177/2054358119827525 30792872 PMC6376545

[13] Goldstein SL Kirkendall E Nguyen H Schaffzin JK Bucuvalas J Bracke T. Electronic health record identification of nephrotoxin exposure and associated acute kidney injury. Pediatrics. (2013) 132:e756–67. 10.1542/peds.2013-0794 23940245

[14] Goldstein SL Dahale D Kirkendall ES Mottes T Kaplan H Muething S. A prospective multi-center quality improvement initiative (NINJA) indicates a reduction in nephrotoxic acute kidney injury in hospitalized children. Kidney Int. (2020) 97:580–8. 10.1016/j.kint.2019.10.015 31980139

[15] Schaffzin JK Dodd CN Nguyen H Schondelmeyer A Campanella S Goldstein SL. Administrative data misclassifies and fails to identify nephrotoxin-associated acute kidney injury in hospitalized children. Hosp Pediatr. (2014) 4:159–66. 10.1542/hpeds.2013-0116 24785560

[16] Xu X Nie S Zhang A Mao J Liu HP Xia H. Acute kidney injury among hospitalized children in china. Clin J Am Soc Nephrol. (2018) 13:1791–800. 10.2215/CJN.00800118 30287424 PMC6302328

[17] Menon S Tarrago R Carlin K Wu H Yonekawa K. Impact of integrated clinical decision support systems in the management of pediatric acute kidney injury: a pilot study. Pediatr Res. (2020) 89:1164–70. 10.1038/s41390-020-1046-8 32620006

[18] Wilson FP Martin M Yamamoto Y Partridge C Moreira E Arora T. Electronic health record alerts for acute kidney injury: multicenter, randomized clinical trial. BMJ. (2021) 372:m4786. 10.1136/bmj.m4786 33461986 PMC8034420

